# Disease Type- and Status-Specific Alteration of CSF Metabolome Coordinated with Clinical Parameters in Inflammatory Demyelinating Diseases of CNS

**DOI:** 10.1371/journal.pone.0166277

**Published:** 2016-11-17

**Authors:** Soo Jin Park, In Hye Jeong, Byung Soo Kong, Jung-Eun Lee, Kyoung Heon Kim, Do Yup Lee, Ho Jin Kim

**Affiliations:** 1 The Department of Bio and Fermentation Convergence Technology, BK21 PLUS project, Kookmin University, Seoul, Korea; 2 The Department of Neurology, Research Institute and Hospital of the National Cancer Center, Goyang, Korea; 3 The Department of Biotechnology, Graduate School, Korea University, Seoul, Korea; Washington University, UNITED STATES

## Abstract

Central nervous system (CNS) inflammatory demyelinating diseases (IDDs) are a group of disorders with different aetiologies, characterized by inflammatory lesions. These disorders include multiple sclerosis (MS), neuromyelitis optica spectrum disorder (NMOSD), and idiopathic transverse myelitis (ITM). Differential diagnosis of the CNS IDDs still remains challenging due to frequent overlap of clinical and radiological manifestation, leading to increased demands for new biomarker discovery. Since cerebrospinal fluid (CSF) metabolites may reflect the status of CNS tissues and provide an interfacial linkage between blood and CNS tissues, we explored multi-component biomarker for different IDDs from CSF samples using gas chromatography mass spectrometry-based metabolite profiling coupled to multiplex bioinformatics approach. We successfully constructed the single model with multiple metabolite variables in coordinated regression with clinical characteristics, expanded disability status scale, oligoclonal bands, and protein levels. The multi-composite biomarker simultaneously discriminated four different immune statuses (a total of 145 samples; 54 MS, 49 NMOSD, 30 ITM, and 12 normal controls). Furthermore, systematic characterization of transitional metabolic modulation identified relapse-associated metabolites and proposed insights into the disease network underlying type-specific metabolic dysfunctionality. The comparative analysis revealed the lipids, 1-monopalmitin and 1-monostearin were common indicative for MS, NMOSD, and ITM whereas fatty acids were specific for the relapse identified in all types of IDDs.

## Introduction

The spectrum of the inflammatory demyelinating diseases (IDDs) of the central nervous system (CNS) includes multiple sclerosis (MS), neuromyelitis optica spectrum disorder (NMOSD), idiopathic transverse myelitis (ITM) and various other inflammatory syndromes. MS is the most common autoimmune demyelinating disease affecting CNS and characterized by periods of relapse and remission, which over time can evolve into a progressive course with accumulating disability. NMOSD has been classified as a subtype of MS for many years. However, the discovery of disease-specific antibodies (NMO-IgG) and subsequent identification of its target, aquaporin-4 (AQP4) [1, 2], led to this view being revised. NMOSD is now considered a distinct disease rather than a variant of MS. Although acute myelitis often occurs in the context of MS or NMOSD, in some cases, no cause can be found for myelitis. ITM is a term used to describe inflammation of the spinal cord after excluding various causes, such as vascular, compressive, metabolic, infectious, and rheumatological disorders [3, 4]. The differential diagnosis of MS, NMOSD and ITM is crucial since the clinical and radiological manifestation of these diseases often overlap. Moreover, the expansion of immunotherapies for MS or NMOSD has generated a need for biomarkers to monitor treatment response. For these reasons, there is an urgent need for the novel biomarkers for differential diagnosis and prognosis.

CSF is critical biological fluid and has unique biological role, including protection against physical damage and nutrient transport for the CNS [5]. The CSF reflects the abnormal immune responses in the CNS more closely than any other biofluids and it provides a unique linkage between the blood and CNS tissue [6]. Thus, investigation of the CSF is an important research tool despite not being easily obtainable compared with other types of biofluids (e.g., blood and urine). Along with well-established CSF inflammatory markers, such as increased IgG synthesis, the presence of oligoclonal bands (OCBs), elevated leukocyte counts and protein levels, identification of the CSF metabolome may insights into the biochemical characteristics of CNS IDDs.

In this study, we applied metabolomics, i.e., the systematic investigation of small molecules, for exploring potential biomarkers and improving our understanding of biochemical features of CSF-mediated autoimmune inflammatory diseases of the CNS. Because of recent advancements in high-throughput molecular profiling technologies, which have permitted sensitive monitoring of the relationship between biofluid/tissue metabolism and pathology [7–10], we used mass spectrometry-based non-targeted metabolite profiling of 145 CSF samples of patients and controls, systematically determined a universal biomarker composite which allowed simultaneous discrimination of each IDD from others including normal control group. We further mechanistically elucidated the biochemical traits of the remission-relapse transition status common in all types of IDDs (MS, NMOSD, and ITM) and disease type-specific metabolic consequences.

## Materials and Methods

### Patient information and clinical manifestations

CSF samples from patients on National Cancer Center registry for inflammatory diseases of the CNS were consecutively collected from May 2005 to April 2015. Available CSF samples from 145 patients suspected to have IDDs were analyzed. Control CSF samples were obtained from 12 healthy subjects who underwent lumbar puncture to rule out meningitis, but turned out to have no medical or neurological illness. All CSF samples were stored at -80°C until use. The demographic and clinical data of these patients, including age, gender, dates of sampling, and Expanded Disability Status Scale (EDSS) score, were collected retrospectively with information regarding disease status (relapse/remission). Diagnoses of MS or NMOSD were based on the 2010 McDonald criteria [11] and the 2015 International Panel for NMO Diagnosis criteria, respectively [12]. Isolated myelitis was defined as idiopathic myelitis after excluding vascular, compressive, infectious, rheumatological, paraneoplastic and radiation-related disorders [3, 4]. Clinical characteristics and CSF profile are summarized in S1 Fig. This study was approved by the research ethics committee of the National Cancer Center. All the procedures were carried out in accordance with the Institutional Review Board of National Cancer Center (NCC2014-0416), and written informed consent was obtained from all subjects.

### Sample preparation

The extraction process, derivatization, and mass-spectrometry analysis were performed for all samples in randomized order.

#### Extraction

CSF samples were thawed on ice at 4°C, and 100μL was mixed with 650μL of extraction solvent (methanol:isopropanol:water, 3:3:2, v/v/v). The mixtures were sonicated for 5 minutes and centrifuged for 5 minutes at 13,200 rpm at 4°C, and 700μL of each supernatant was aliquoted into a new 1.5-mL tube. The aliquots were concentrated to complete dryness in a speed vacuum concentrator (SCANVAC, Korea). Dried extracts were stored at -80°C until derivatization and gas chromatography time-of-flight mass spectrometry (GC-TOF MS) analysis.

#### Derivatization

The dried extracts were mixed with 5 μL of 40 mg/mL methoxyamine hydrochloride (Sigma-Aldrich, St. Louis, MO, USA) in pyridine (Thermo, USA) and then incubated for 90 min at 200 rpm and 30°C for methoxyamination. After the first derivatization process, 2 μL of a mixture of internal retention index (fatty acid methyl esters [FAMEs]) and 45 μL of *N*-methyl-*N*-trimethylsilyltrifluoroacetamide (MSTFA + 1% TMCS; Thermo, USA) were added for trimethylsilylation (1h at 200 rpm and 37°C). The FAME mixture was composed of C8, C9, C10, C12, C14, C16, C18, C20, C22, C24, C26, C28, and C30.

### GC-TOF MS analysis

The derivatives (0.5 μL) were injected using an Agilent 7693 ALS (Agilent Technologies, Wilmington, DE, USA) in splitless mode. Chromatographic separation was carried out using an Agilent 7890B gas chromatograph (Agilent Technologies) equipped with an RTX-5Sil MS column (Restek, Gellefonte, PA, USA). The oven temperatures were programmed at 50°C for 1 min, ramped at 20°C/min to 330°C, and held constant for 5 min. Mass spectrometry analysis was performed on a Leco Pegasus HT time of flight mass spectrometer controlled by ChromaTOF software 4.50 version (LECO, St. Joseph, MI, USA). Mass spectra were acquired in the mass range of 85–500 *m/z* at an acquisition rate of 17 spectra/s.

Raw result files were collected and pre-processed using ChromaTOF software, and further process using *Binbase*, an in-house database. ChromaTOF-specific peg files were converted to generic *.txt result files and additionally as generic netCDF files for further data evaluation. More details can be found in previous reports [13, 14]. A total of 85 out of 962 metabolic signals were finally reported with occurrence in 50% of the samples per study design group. The quality control was carried out with mixture of 31 pure reference compounds between every 10 samples [13, 15, 16].

### Statistics, data visualization, and metabolic network construction

Quantitative mass spectrometry (providing a unique m/z value) for each chemical was selected according to the algorithm [14], and the peak height was used for relative quantification. The semi-quantitative values were then normalized to the sum intensity of identified peaks of each chromatogram for quantitative comparison. General statistics including t-tests was conducted on all continuous variables using *Statistica* software version 7.1 (StatSoft, Tulsa, OK, USA). K-mean clustering analysis and heatmap visualization using Spearman rank correlation and average linkage methods was done within *Multi Experimental Viewer* (MeV, TIGR) [17]. Multivariate statistical analysis was performed on partial least squares discriminant analysis (PLS-DA) and orthogonal partial least squares discriminant analysis (OPLS-DA) were performed in *SIMCA 14* (Umetrics AB, Umea, Sweden). Receiver operating characteristic (ROC) and SUS plot analyses was carried using the appropriate modules implemented in *SIMCA 14*. The 95% confidence interval using bootstraping for ROC analysis was performed using *Biomarker analysis* panel implemented in *MetaboAnalyst* 3.0 [18, 19].

Metabolic network construction was carried out as previously reported [15, 20–22]. Briefly, compound identifiers (CIDs) were collected for all identified metabolites, and *Tanimoto* scores were calculated from PubChem Compound database. A threshold *Tanimoto* score of 0.7 was applied for the connectivity cut-off value for each metabolite pair. The connectivity matrix was overlaid by additional edges composed of metabolite pairs satisfying the KEGG Rpair reaction (substrate-product relation). The two network correlation matrices were imported into the Cytoscape interface [23] as edge information (simple interaction format, SIF file) with separate node information (metabolite information). The network was visualized in an organic layout with some modifications for clear imaging. Fold change was presented as node size, and direction (up/down) was imaged as node color (red/blue resp.) only for metabolites passing the statistical criteria (Student’s t-tests, *P*< 0.05). Pathway analysis was carried out using *Metaboanalyst* where significant levels were estimated based on hypergeometric test and pathway impact values were assessed by relative-betweenness centrality [18].

## Results and Discussion

### CSF metabolome mirrors differential metabolic dysfunction triggered by autoimmune disorders

Mass spectrometry-based metabolite profiling was performed for a total of 145 CSF samples, (MS = 54, NMOSD = 49, ITM = 30 and normal controls = 12). Of the 962 metabolic signatures (known + unknown) that passed the criteria described in the method, 85 were structurally identified and quantified by GC-MS. Data can be downloaded online [https://lms2.kookmin.ac.kr:446/index.php?hCode=PAPER_LIST&publication_name=inter_paper] or [https://figshare.com/s/5a5e934a64f151b03b47], with the full description of all detected peaks, identified metabolites, retention indices, mass spectra, and quantification ions. The identified metabolites were classified as sugars and sugar alcohols (24%), amino acids (28%), fatty acids (15%), organic acids (15%), amines (2%), phosphates (1%), and miscellaneous compounds.

Univariate statistics were initially used to analyze compositional changes in metabolites associated with autoimmune disorders (three disease groups vs the control). A total of seven metabolites were significantly changed (Student’s t-test *P*<0.05, Table 1) in the autoimmune disease groups compared to those in the reference group.

**Table 1:** Univariate statistics for metabolites significantly altered in the diseases.

**Control vs MS**			**Control vs NMOSD**		
**Compounds**	***p*-value**	**Fold change**	**Compounds**	***p*-value**	**Fold change**
1-monostearin	3.30E-09	1.72	1-monostearin	2.97E-05	1.97
glycolic acid	1.70E-02	1.65	1-monopalmitin	2.48E-05	1.60
1-monopalmitin	2.23E-06	1.43	salicylaldehyde	1.01E-02	1.53
phenylalanine	8.01E-03	0.84	fumaric acid	3.93E-02	1.21
tyrosine	1.71E-02	0.80	lactic acid	4.54E-02	1.11
3-hydroxypropionic acid	1.56E-02	0.79	3-hydroxypropionic acid	3.63E-03	0.78
inosine	1.39E-03	0.74	inosine	2.70E-03	0.74
valine	3.30E-03	0.70	threose	4.08E-10	0.39
leucine	4.21E-03	0.69	butane-2,3-diol	3.13E-02	0.19
isoleucine	1.34E-03	0.66			
proline	5.65E-03	0.61			
methionine	1.80E-02	0.39			
threose	2.30E-11	0.36			
butane-2,3-diol	3.19E-02	0.22			
**Control vs ITM**			**Control vs All diseases**		
**Compounds**	***p*-value**	**Fold change**	**Compounds**	***p*-value**	**Fold change**
1-monopalmitin	2.74E-04	1.48	1-monostearin	4.37E-06	1.82
1-monostearin	3.53E-04	1.75	1-monopalmitin	8.01E-06	1.50
lactic acid	1.36E-02	1.15	glycolic acid	4.52E-02	1.44
hypoxanthine	1.52E-02	1.34	inosine	8.22E-03	0.78
glutamine	2.55E-02	1.44	glycine	3.67E-02	0.66
benzoic acid	4.38E-02	1.17	threose	2.08E-06	0.47
			butane-2,3-diol	1.07E-03	0.23

**Fig 1:**
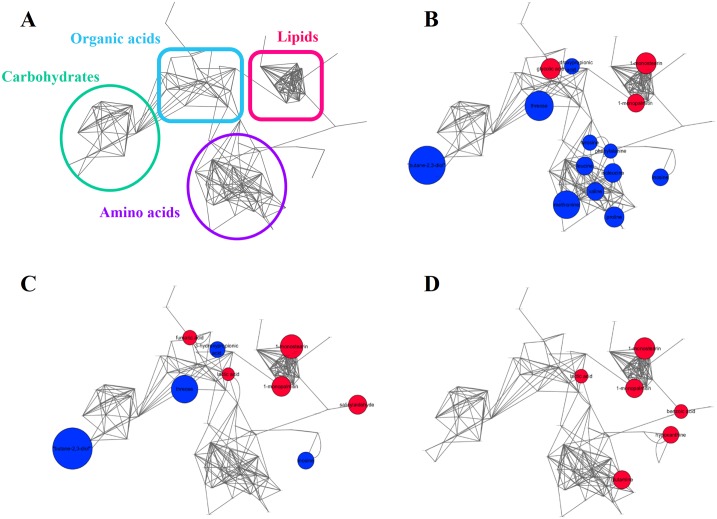
Distinct IDDs anchorage showed “signature” pools of primary CSF metabolites. (A) Schematic representation of chemical categories of metabolites from network analysis and the disease metabolic networks of (B) MS, (C) NMOSD, and (D) ITM. The edges of the metabolic networks were composed of chemical structure similarity and Kegg reaction pairs (gray). Node color presents up- (red) and down-regulation (blue) compared to the ones in control, and node size indicates fold changes (disease/control) among identified metabolites. Metabolites that were not significantly different were omitted for visual clarity.

1-monopalmitin, 1-monostearin, and glycolic acid were up-regulated whereas glycine, inosine, threose, and butane-2,3-diol were decreased in the disease group. Next, pair-wise statistical comparisons between each autoimmune disease and the reference group revealed disease-specific alteration in CSF samples. To systematically capture a comparative view of unique metabolic consequences mirrored in CSF metabolome, we applied a multi-layered metabolic network analysis approach that could provide an integrative view of metabolic modulation at the level of a biochemical entity (Fig 1A). Primarily, the metabolic network analysis revealed the systematic alteration of amino acids in MS, where tyrosine, phenylalanine, leucine, isoleucine, valine, methionine, and proline were significantly decreased while other types of IDDs (NMOSD and ITM) showed the similar levels compared to normal control (Fig 1B). The unique metabolic phenotype in MS was in accordance with previous studies [24, 25]. Particularly, aromatic amino acids, phenylalanine and tyrosine showing lower levels in MS have been used solely or in combination with other compounds as an effective drug to relieve MS symptoms [26, 27]; the mechanism of action may be associated with increased synaptic availability of the neurotransmitter [28].

The NMOSD disease network exhibited similar alterations in carbohydrate and organic acid modules (e.g., threose, 3-hydroxypropionic acid and butane-2,3-diol), with exception of the increased central metabolic components such as fumarate [29, 30] and lactate [31] (Fig 1C). Morishima *et al*. reported that lactic acid-induced CNS acidosis may cause the increased expression and altered distribution of AQP4 in astrocytes [32]. The increases in AQP4 membrane expression during acute attacks could potentially enhance the complement-mediated humoral immune reaction against AQP4 expression in astrocytes, leading to more severe astrocytic damage. Interestingly, one CSF study showed that lactate levels were elevated in CSF samples obtained during acute attacks compared with those during remission [33]. Based on these findings, CSF lactic acidosis may potentially play a role in the pathophysiology of seropositive NMOSD [34]. In comparison, metabolic disruptions in ITM were best characterized by significant up-regulation of all metabolites (Fig 1D). Benzoic acid, hypoxanthine, and glutamine were ITM-specific metabolic changes while lactic acid, 1-monopalmitin, and 1-monostearin were common features found in all types of autoimmune disorders. Of particular interest, 1-monopalmitin and 1-monostearin were the only lipid components that were significantly altered (upregulated) in all disease states when compared with the appropriate references, which may be linked to the cellular toxicity in common across different types of autoimmune inflammatory disorders of the CNS [35, 36].

### Single biomarker cluster for discriminating multiple disease type in combination of CSF metabolite and clinical characteristics

Prior to biomarker screening, we inspected any substantial differences in the CSF metabolome influences by clinical therapies (e.g. interferon therapy for MS and immunosuppresive therapy for NMOSD and ITM). The score scatter plot using unsupervised multivariate statistics (principal component analysis) showed no significant segregation by the treatment effect (S2 Fig).

**Fig 2:**
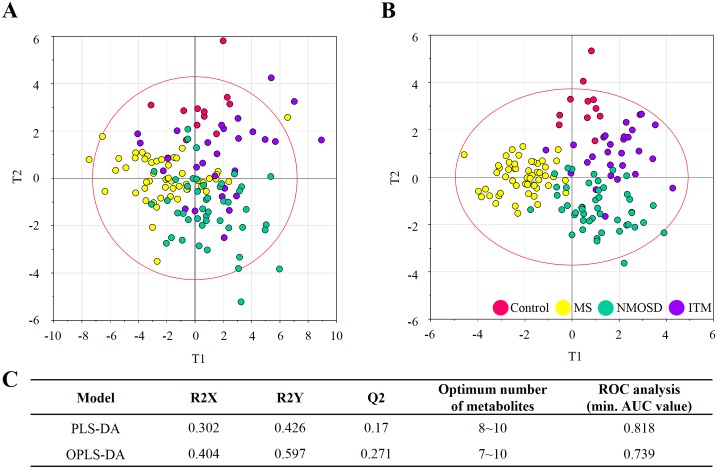
Exploration of disease type-specific biomarkers using multivariate statistical models coupled ROC analysis. Score scatter plots of (A) PLS-DA and (B) OPLS-DA models. T1 indicates the discriminating vector 1, which explained the largest degree of variation in the dataset and T2 indicates principal component 2 in PLS-DA and OPLS-DA respectively. (C) Cumulative goodness-of-fit (R^2^Y), predictability (Q^2^), evaluation of selected metabolites by ROC analysis.

Supervised multivariate statistical analysis were performed to inspect whether the integrative metabolomic phenotypes could be distinguished, potentially leading to biomarker discovery for the different types of IDDs with robust modelling and validation processes. PLS-DA and OPLS-DA were applied to explore the universal discriminant factors that ideally separate the metabolite profiles of each immune disorder (references, MS, NMOSD, and ITM) against each other. Among them was OPLS-DA model which showed the best cumulative goodness-of-fit and predictive ability (R^2^Y = 0.597 and Q^2^Y = 0.271) (Fig 2).

**Fig 3:**
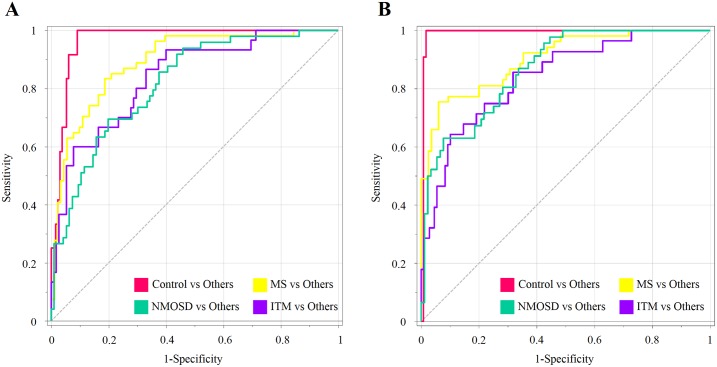
ROC analysis and resulting AUC values of multiple-metabolite panels from the CSF in three different autoimmune disorders. **(A)** The area under the curve (AUC) are for biomarker panel, which consisted of only CSF metabolites (threose, lactic acid, 1-monostearin, 1-monopalmitin, 3-hydroxypropionic acid, inosine, threitol, phenylalanine) and (B) biomarker panel, which was coordinately integrated with three clinical parameters (threose, EDSS, lactic acid, 1-monostearin, 1-monopalmitin, OCB, 3-hydroxypropionic acid, protein levels, inosine, phenylalanine).

Next, we explored a candidate biomarker panel which could simultaneously discriminate three different types of CNS IDDs. In addition to the primary goal, biomarker selection, we examined the minimal number of metabolites for the biomarker panel conceiving the practicality of the selected metabolites for clinical applications. Considering potential end application (e.g. clinics) and platform-cross validation, the assessment of biomarker performance was carried out using ROC curve analysis, which is generally regarded as the standard for describing and evaluating the performance of medical diagnostic tests [37]. Accordingly, high-impact metabolites for the discrimination were prioritized using the variable importance in the projection (VIP). The abundances of selected metabolites were coordinately transformed to a single component (score matrix, T1) using each multivariate statistical algorithm for numerical evaluation using ROC analysis. The best result was obtained from PLS-DA model where biomarker composites with 8–10 metabolites reached the maximal area under the curve (AUC) vales for all comparison (S3 Fig). The primary composite with 8 metabolites was composed of threose, lactic acid, 1-monostearin, 1-monopalmitin, 3-hydroxypropionic acid, inosine, threitol, phenylalanine. The recomposed panel achieved AUCs that ranged from 0.818 to 0.967 in all diagnostic sets (Fig 3A). The AUCs of control-others, MS-others, NMOSD-others, and ITM-others were 0.967, 0.894, 0.818, and 0.839 respectively. In addition, we examined if other CSF markers or their combination could improve the model power in coordination with the CSF metabolite biomarkers indicators prioritized based on PLS-DA. Thus four CSF markers and one clinical parameter available in the current study, white blood cell counts, protein levels, oligoclonal bands (OCB), IgG index, and expanded disability status scale (EDSS) were evaluated and selectively employed in a new multivariate biomarker model. The EDSS, OCB, and protein levels were chosen based on VIP scores, and the resulting joint model with the recombined top 10 factors (seven metabolites + three CSF parameters) improved the AUC with the values of 0.991, 0.904, 0.869, and 0.842 (control-others, MS-others, NMOSD-others, and ITM-others respectively) (Fig 3B). The result indicated the coordinated matrix integrated with CSF characteristics enhanced the diagnostic power for the multi-comparison of the predictive model. OCB is often used as a supportive diagnostic and poor prognostic marker, exhibiting relatively high specificity for MS, therefore, OCB has potential prognostic value and is helpful for clinical decision-making [38]. Additionally, the elevated protein levels, although not a confirmative diagnostic parameter for MS when used alone, indicate an abnormal immune response. Among the clinical characteristic were EDDS that was top-ranked based on VIP score. EDSS is considered the standard measurement of the disability grade of patients with MS, which was invented to objectively quantify the functioning levels of MS patient with total score ranged from 0 to 10 [39]. The 95% confidence intervals were re-computed using 500 bootstrappings after extracting the biomarker component (T1) and employing to *Biomarker analysis* module implemented in *MetaboAnalyst* 3.0, which is summarized in S4 Fig.

**Fig 4:**
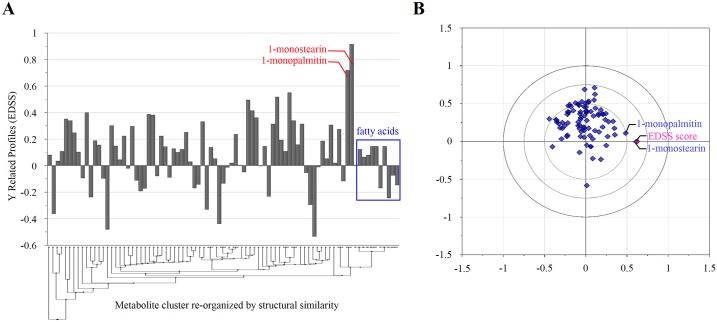
Identification of CSF metabolites mainly contributing to EDSS in OPLS model. (A) The metabolites positively and negatively correlated with EDSS were shown in Y-related profile where all identified metabolites and EDSS were set to x and y blocks, respectively. X-axis was re-organized according to structural similarity among metabolites. (B) Loading scatterplot exhibiting the relative coherence between the CSF metabolites and EDDS.

We further explored any underlying relatedness between the disability and CSF metabolome, which may provide more objective monitoring criteria for the patients. In order to extract pure estimate of the underlying constituents in x variables (CSF metabolites) corresponding to EDSS, we performed Y-related profiles using OPLS model where all metabolites and EDSS were set to x and y blocks respectively. 1-monostearin and 1-monopalmin exhibited the strongest correspondence to EDSS in the profile (Fig 4A), consistent with the loading scatter plot (Fig 4B). Interestingly, the substantial contribution of the uncommon lipids to EDSS was contrast to relatively minor influence of various types of fatty acids. The monoacylglycerols have been investigated for potential implication in dysregulated thyroid [36], which often presents comorbidities with other types of autoimmune disorders [40] and makes an attribution to common disabling symptom in MS [41]. Associated with the commonality in their significant alteration across all types of the diseases and the major contribution to the biomarker panel for simultaneous discrimination of each disease type, these uncommon lipids could be new diagnostic and prognostic molecular indicators, and therapeutic targets for IDDs. The strongest negative correlation with EDSS was found in ethanolamine, threose, and 1,5-anhydroglucitol.

### Characteristic metabolic switching between relapse and remission status

**Fig 5:**
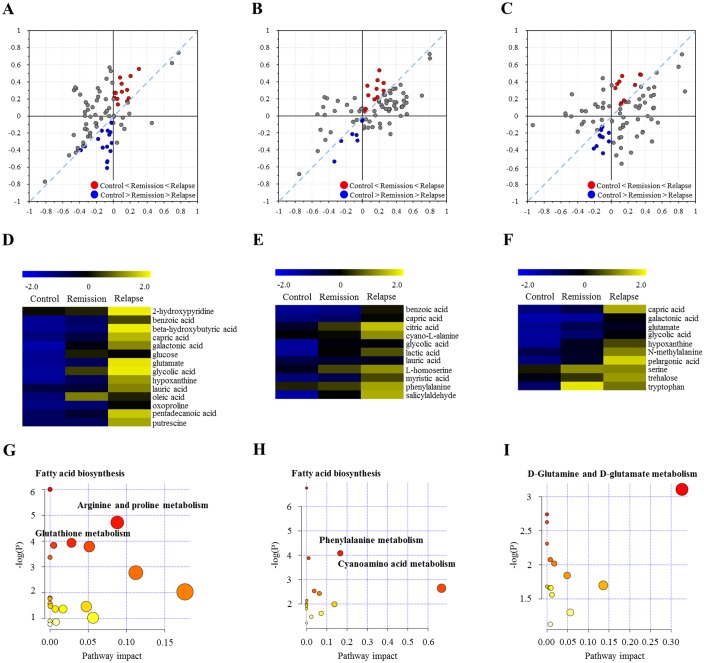
Shared-and-unique-structures (SUS) plots with the p(corr) values in the OPLS-DA model. The SUS plot analyzed the relative contribution of metabolite variables to the discriminant models of control versus remission (X-axis) and control versus relapse (Y-axis) in (A) MS, (B) NMOSD, and (C) ITM. The variables located near from the X-axis and Y-axis indicate remission- and relapse-specific metabolites, respectively. The metabolite variables close to the diagonal represent the common expression pattern in remission and relapse compared with that in the control. The metabolites positioned in upper region (red) from the diagonal showed gradual increase in abundance in the order of control < remission < relapse, corresponding to the pathological status. The metabolite screening from SUS plot analysis was confirmed by K-mean clustering analysis for (D) MS, (E) NMOSD, and (F) ITM, respectively. (G), (H), (I) present the overview of pathway analysis for MS, NMOSD, and ITM respectively. X-axis indicates pathway impact values (based on relative-betweeness centrality), and Y-axis presents *P*-values (based on hypergeometric test).

Understanding the transition status between remission and relapse and identifying relapse-associated metabolites is important diagnostic and prognostic application. However, no confirmative biomarkers have been identified in biological fluids that are capable of monitoring disease status of CNS IDDs. Accordingly, we speculated the metabolite changes corresponding to remission and relapse statuses only with the CSF samples taken before the initiation of high dose methylprednisolone (57 and 61 CSFs for remission and relapse respectively). To isolate metabolite patterns corresponding to the relapse specificity, we applied shared-and-unique-structures (SUS) plots with p(corr) values in the OPLS-DA model. The SUS plot showed the metabolite variables, which were coordinately or uniquely contributing to relapse and remission in MS, NMOSD, and ITM respectively. The plot systematically isolated the sets of metabolites showing gradual changes, which could be crucial determinants of the remission-relapse transition (Fig 5). The metabolites positioned in upper region (red) from the diagonal showed up-regulation in both remission and relapse compared to the references, and concurrently exhibited higher levels in relapse than in remission, which implied the clinical aggravation among the different stages (control < remission < relapse). The SUS plot showed good cumulative goodness-of-fit and the moderate predictive ability in the models. Following the screening of stage-specific metabolites based on the SUS plots, the expression pattern was validated by K-mean clustering analysis (Fig 5D–5F).

Subsequent pathway over-representation analysis for MS suggested that 14 metabolites with gradual increases in MS (control < remission < relapse) were enriched for fatty acid and arginine-proline metabolisms in MS-relapse (Fig 5A, 5D, and 5G). The metabolites exclusively specific for MS-relapse were 2-hydroxypyridine, beta-hydroxybutyric acid, glucose, pentadecanoic acid, oleic acid, oxoproline, and putrescine. Likewise, the relapse stage of NMOSD showed consensus metabolic dysregulation in fatty acid metabolism consistent with MS (Fig 5B, 5E, and 5H). In addition, CSF metabolites indicative of the relapse specificity for NMOSD were cyano-l-alanine, lactic acid, citric acid, homoserine, phenylalanine, myristic acid, and salicyladehyde (Fig 5B and 5E). Note that cyano-l-alanine is an inhibitor of cystathione γ-lyase (CSE), producing H_2_S, which has been recently proposed as a new neuromodulator with functional roles in inflammation control [42], and as an anti-oxidant in the brain [43]. The disrupted metabolism specific for the relapse of NMOSD was further linked to the synchronized alteration of phenylalanine and homoserine in sulfur metabolism. In the context of ITM, the relapse was distinctively characterized by gradual increases in N-methylalanine, pelargonic acid, trehalose, serine, and tryptophan. The implication of potential neuro-toxicity has been reported in association with the increased levels of N-methylalanine and pelargonic acid, which disturbs CNS homeostasis and inhibit biotin transfer through blood-brain barrier respectively [44, 45].

In addition to the relapse-specific alterations in each disease, we further examined the pathological co-existence of CSF metabolites according to relapse-remission comparison regardless of the disease type in merged dataset. Resultant 16 metabolites, presenting a gradual increase in the order of relapse > remission > control, were mainly classified as fatty acids (fatty acid derivatives; S5 Fig). Univariate statistics confirmed the significant up-regulation of most of the fatty acids under the relapse state compared with that during remission. A few studies have reported the potential therapeutic effects by modulating the endogenous ratio of saturated fatty acids (SFAs) and polyunsaturated fatty acids (PUFAs) with omega-3 and omega-6 supplementation which may moderate immune-cell activation via multiple complex pathways [46–48]. Moreover, a recent report elucidated a fatty acid-associated pathological mechanism and potential therapeutic targets using experimental autoimmune encephalomyelitis (EAE), a mouse model of MS [49]. Similarly, in our study, the aberrant activities of a wide range of fatty acids were monitored, and particularly SFAs were exclusively up-regulated in the relapse state compared with those in remission, which resulted in higher ratio of SFA to PUFA. These fatty acids, ranging from C12 to C18, included capric acid, lauric acid, myristic acid, heptadecanoic acid, and steric acid. The possible dysfunction in β-oxidation was concurrent with higher central energy metabolic activity, as reflected by increased levels of lactic acid and fumarate in the CSF during relapse stage. Other abnormal changes were observed in amino acids and amines primarily associated with arginine-proline metabolism and alanine-aspartate-glutamate metabolism. It is intriguing that the significant alteration in SFAs were not identified in control-disease comparison, but corresponded to the relapse-specific modulation, which suggests the significance of the metabolism as putative therapeutic targets for symptom alleviation [46, 50].

## Conclusion

Although requiring expansion of observation, successive validation, and additional examination under controlled circumstances eventually for practical application to clinics, the current investigation is one of the largest studies with broad coverage of CSF metabolome, which explores the multi-component biomarker for multiplex CNS IDDs. The biomarker cluster coordinately derived from 7 CSF metabolites and other clinical and CSF parameters (EDSS, OCB, and protein levels) simultaneously discriminated different types of CNS IDDs from reference group and each other. At the same time, we proposed a disease-specific metabolic network, which may improve our understanding of the disease mechanism reflected in CSF metabolome. Finally, we identified the characteristic modulation of the CSF metabolome (e.g., fatty acids) with the transformation of disease status (i.e., relapse-remission). The findings suggest that CSF metabolic profiling can increase the accuracy of diagnosis in different types of CNS IDDs, and may help clinicians in appropriate therapeutic decision-making. Further, the discovery reported in the current study can increase the value in clinical applicability with additional validation ideally using a second cohort study from independent source, and can guide therapeutic plans with more detailed mechanistic understanding from case-control study.

## Supporting Information

S1 Fig Subject and sample characteristics by subject group. (TIF)

S2 Fig Principal component analysis of metabolomic profiles of CSF samples. Score scatter plots show no significant effect of therapeutic treatments regardless of IDDs types.
(TIF)

S3 Fig Optimal number of metabolites in the biomarker panel. The X-axis indicates the number of metabolites within the biomarker panel. To optimize this number, area under the curve (AUC) values were calculated using receiver operating characteristic (ROC) analysis, as depicted on the Y-axis.
(TIF)

S4 Fig ROC curves with the 95% confidence intervals using 500 bootstrappings. (TIF)

S5 Fig Shared-and-unique-structures (SUS) plots with the p(corr) values in the OPLS-DA model. The SUS plot analysis for the relative contribution of metabolite variables to the discriminant models of control versus remission (X-axis) and control versus relapse (Y-axis) in merged data set (control vs all disease).
(TIF)
