# Supplementary figures and images for: Disease Type- and Status-Specific Alteration of CSF Metabolome Coordinated with Clinical Parameters in Inflammatory Demyelinating Diseases of CNS
Source: PLoS One. 2016 Nov 17;11(11):e0166277. doi: 10.1371/journal.pone.0166277 (PMC5113962; doi:10.1371/journal.pone.0166277)

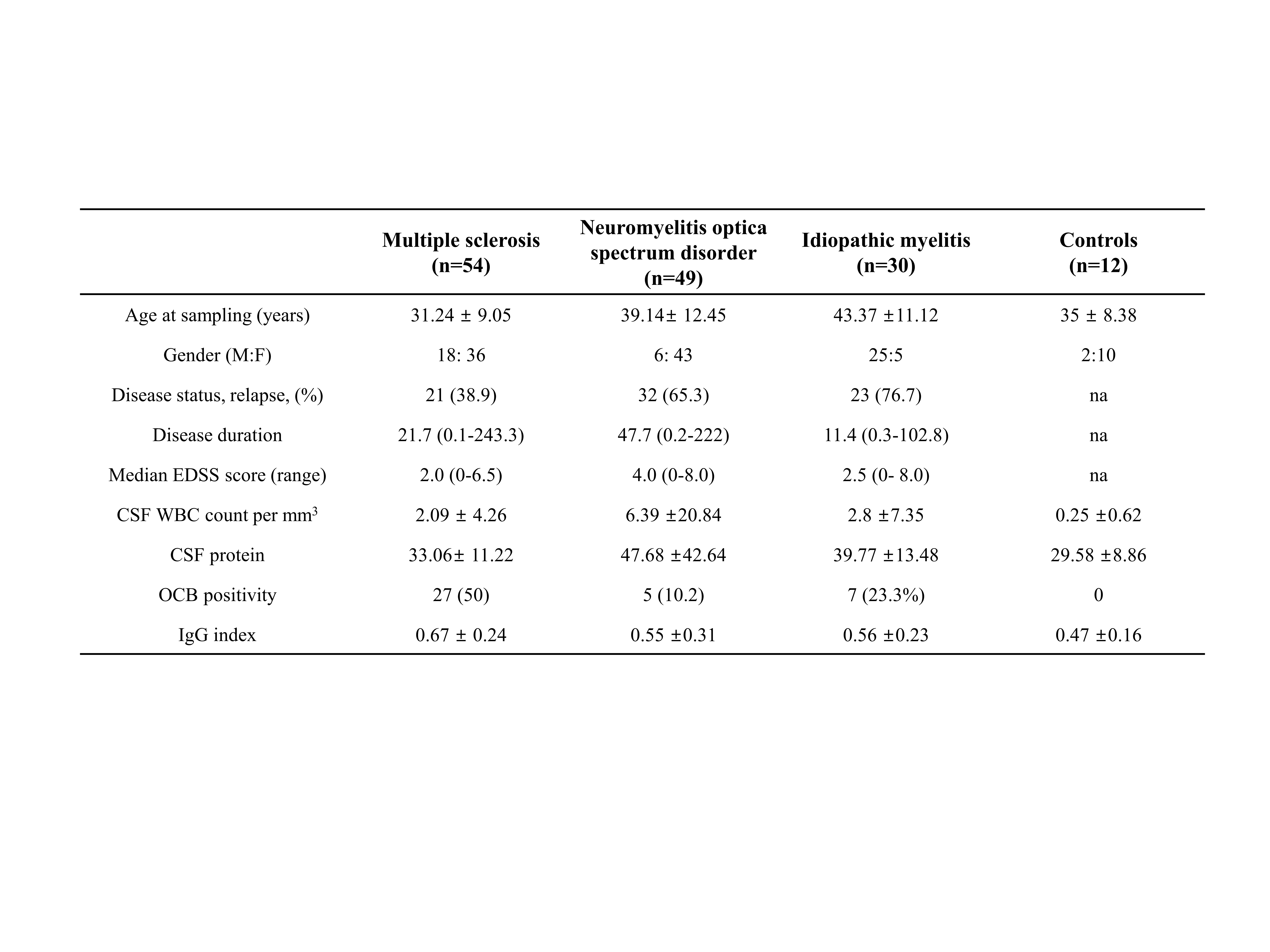

Supplement: S1 Fig — (TIF) [file pone.0166277.s001.TIF]

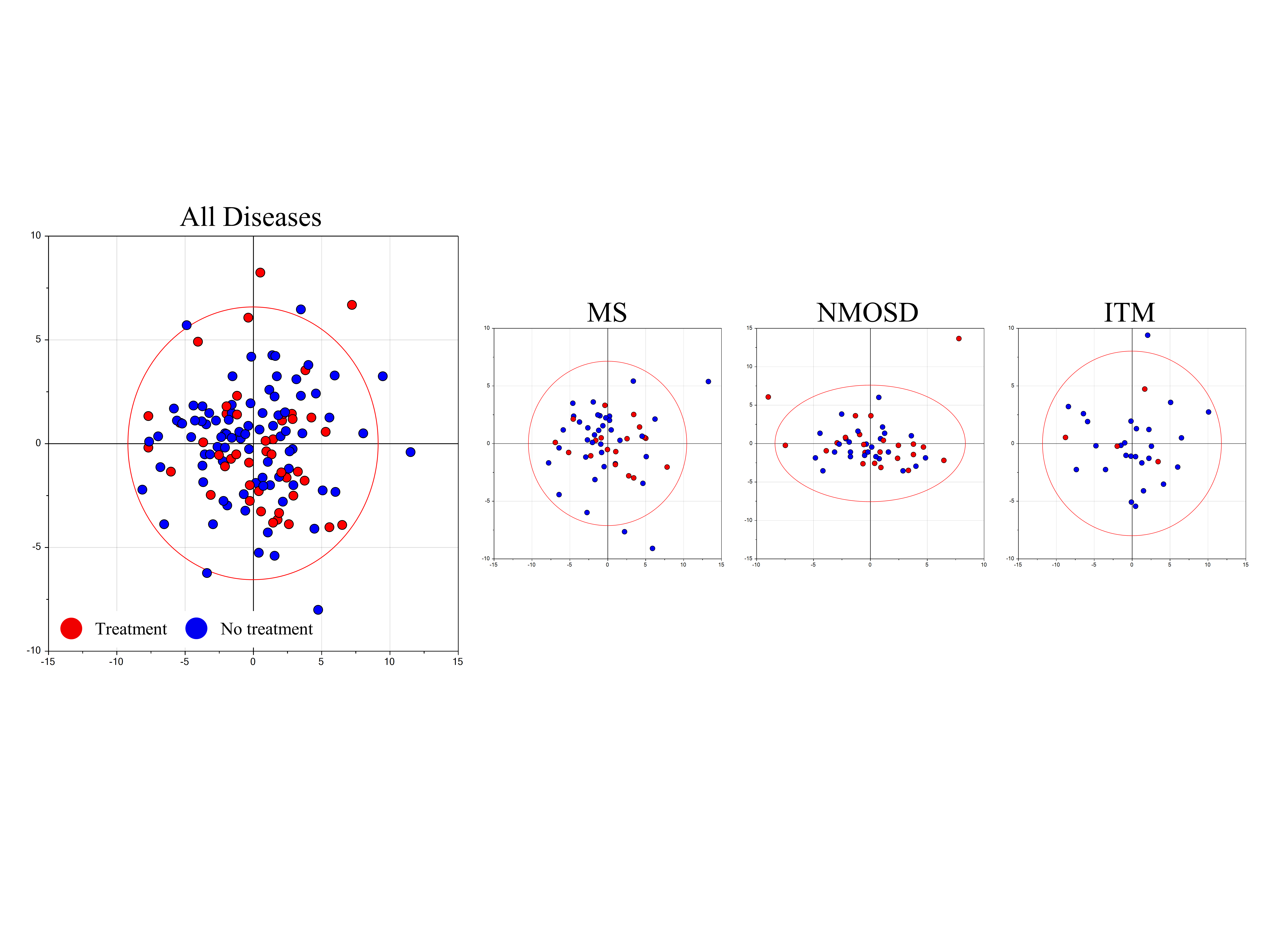

Supplement: S2 Fig — Score scatter plots show no significant effect of therapeutic treatments regardless of IDDs types. (TIF) [file pone.0166277.s002.tif]

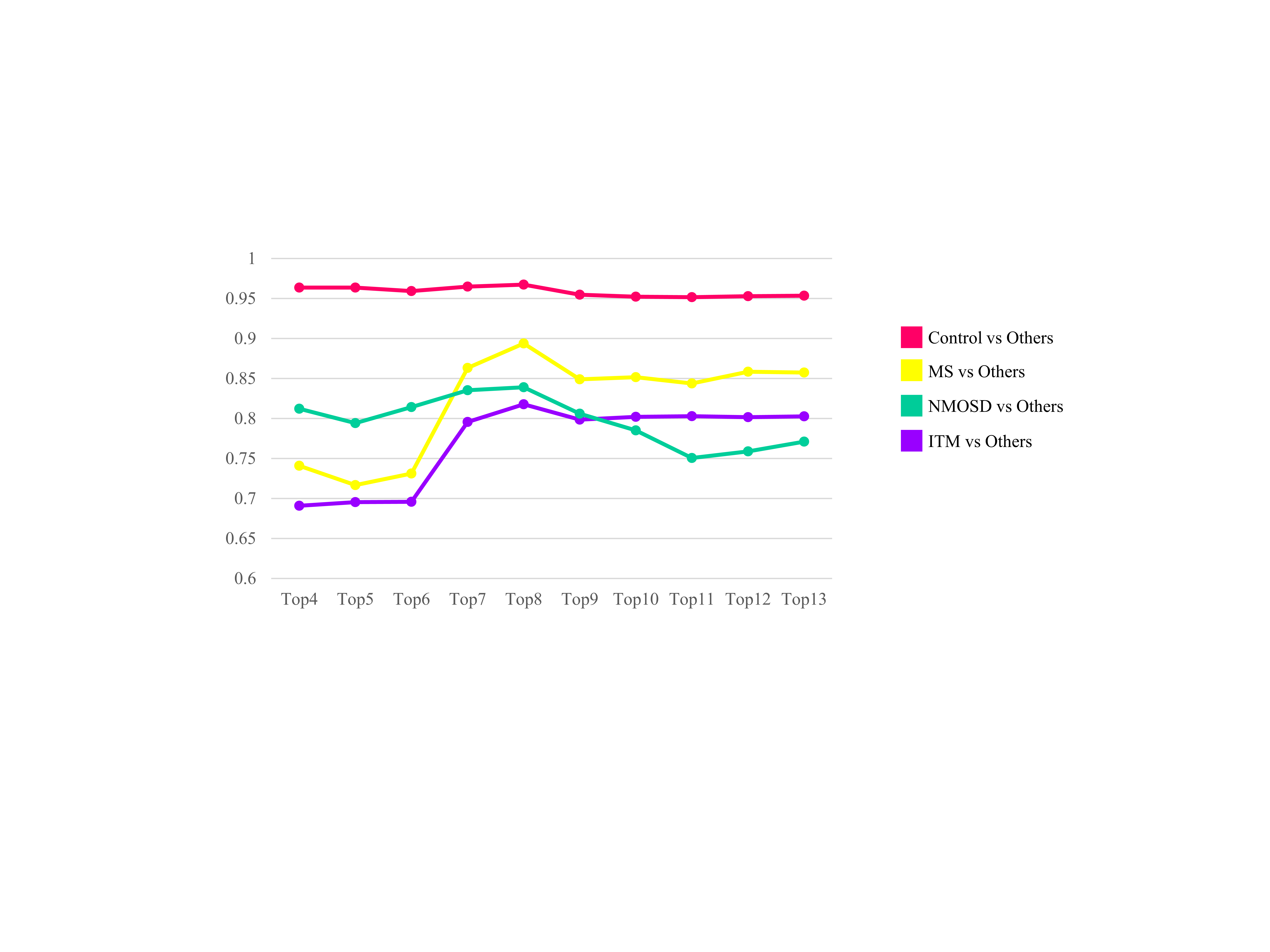

Supplement: S3 Fig — The X-axis indicates the number of metabolites within the biomarker panel. To optimize this number, area under the curve (AUC) values were calculated using receiver operating characteristic (ROC) analysis, as depicted on the Y-axis. (TIF) [file pone.0166277.s003.TIF]

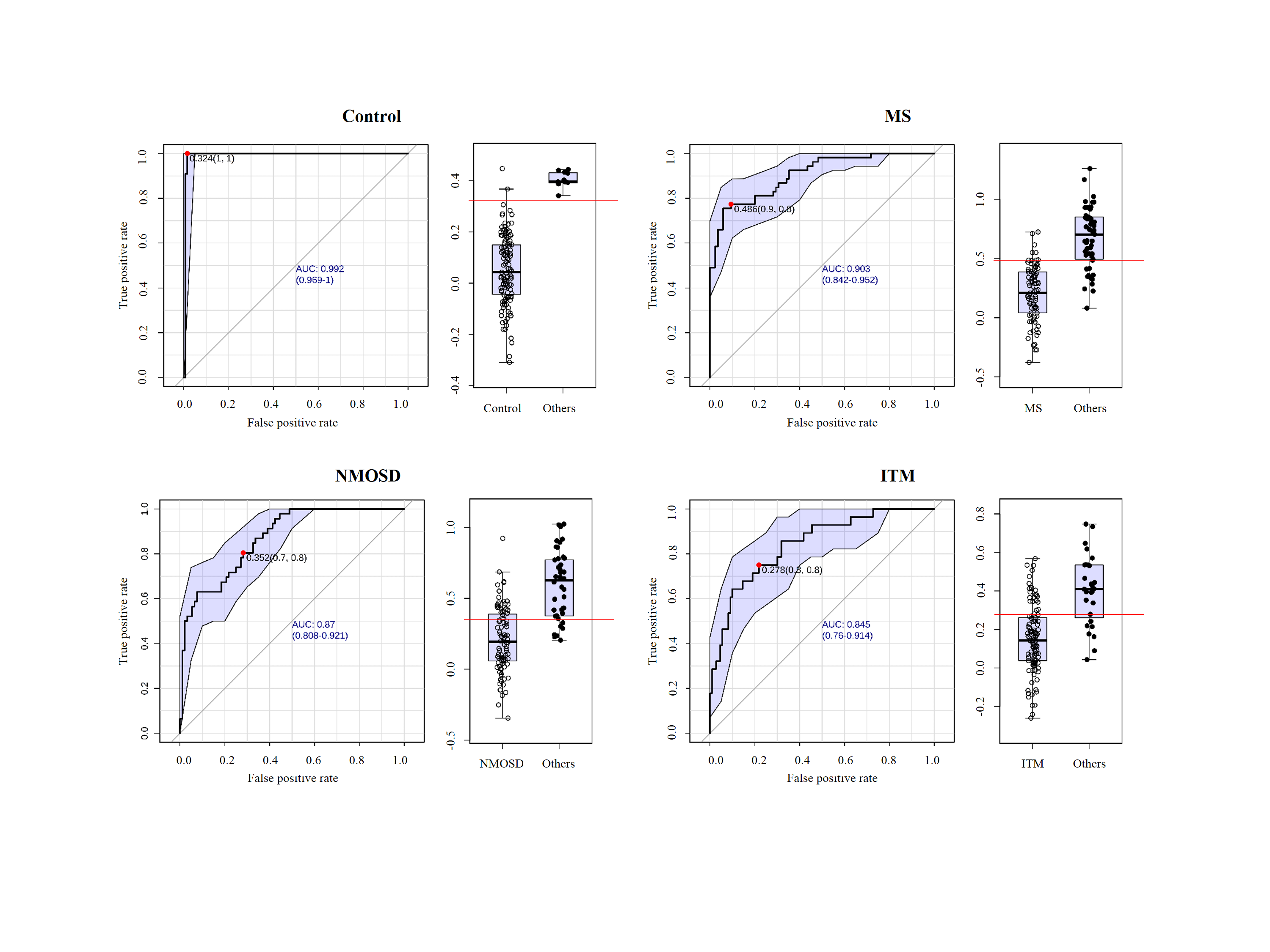

Supplement: S4 Fig — (TIF) [file pone.0166277.s004.tif]

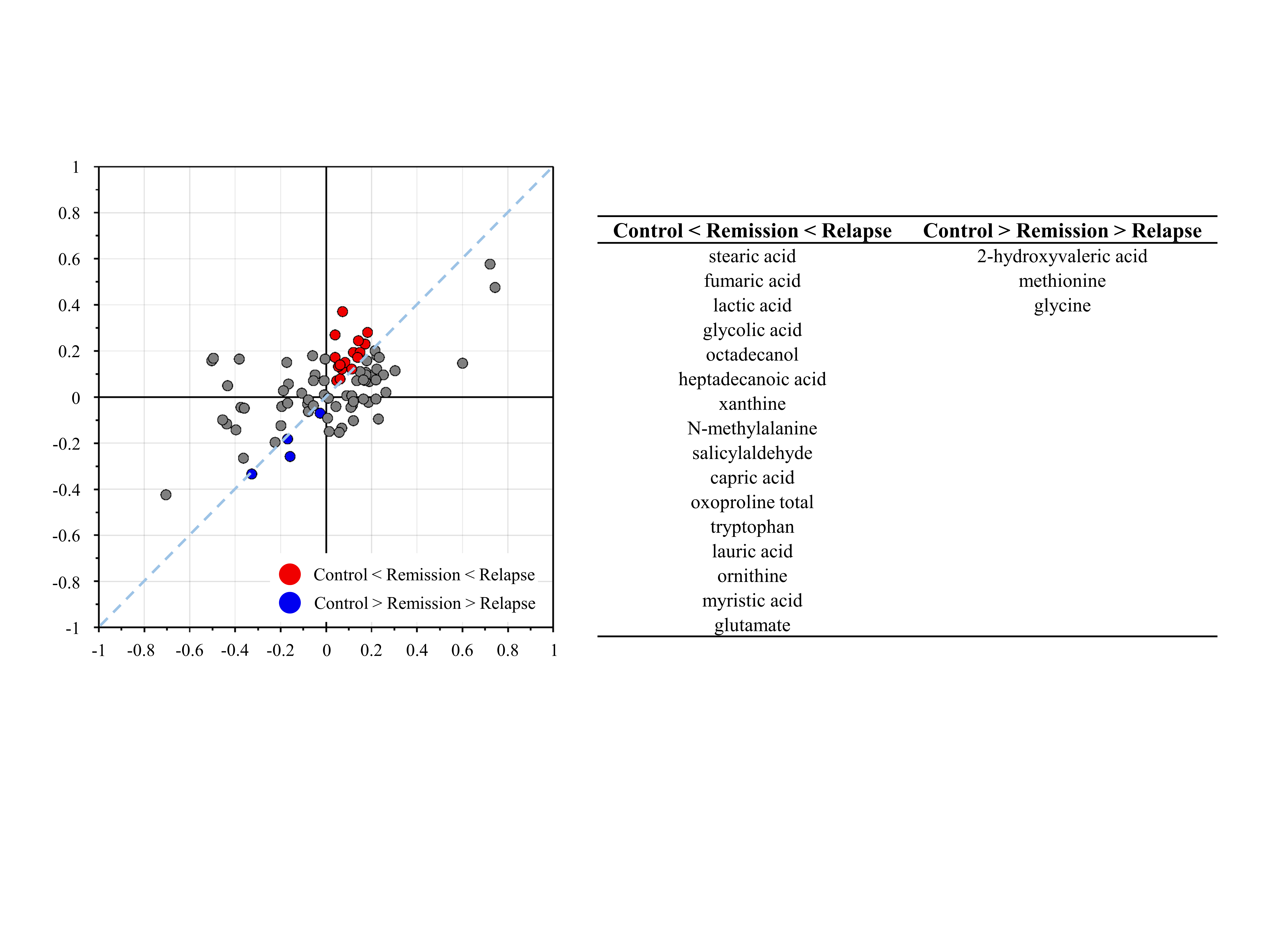

Supplement: S5 Fig — The SUS plot analysis for the relative contribution of metabolite variables to the discriminant models of control versus remission (X-axis) and control versus relapse (Y-axis) in merged data set (control vs all disease). (TIF) [file pone.0166277.s005.TIF]
